# Endoplasmic Reticulum Stress Affects Cholesterol Homeostasis by Inhibiting LXRα Expression in Hepatocytes and Macrophages

**DOI:** 10.3390/nu12103088

**Published:** 2020-10-11

**Authors:** Tian Wang, Yiyang Zhao, Zhongsheng You, Xiatian Li, Mingdi Xiong, Hua Li, Nianlong Yan

**Affiliations:** Department of Biochemistry and Molecular Biology, College of Basic Medical Science, Nanchang University, Nanchang 330006, China; avasweet1213@163.com (T.W.); ashelyzhaoyiyang@163.com (Y.Z.); Sain827771427@163.com (Z.Y.); 15807939939@163.com (X.L.); amourxiong@sina.com (M.X.); lihua880303@163.com (H.L.)

**Keywords:** endoplasmic reticulum stress, LXRα, cholesterol metabolism, atherosclerosis, reverse cholesterol transport

## Abstract

Atherosclerosis (AS) is the most common cardiovascular disease, and reverse cholesterol transport (RCT) plays an important role in maintaining cholesterol homeostasis. Both endoplasmic reticulum (ER) stress and LXRα can affect the metabolism of cholesterol. However, whether ER stress can modulate cholesterol metabolism by LXRα in hepatocytes and macrophages remains unclear. Therefore, in this study, we aimed to explore the relationship between ER stress induced by tunicamycin and LXRα in hepatocytes and macrophages and clarify their possible mechanisms and roles in AS. C57BL/6 mice and Huh-7 and THP-1 cells were treated with tunicamycin and LXR-623 (an agonist of LXRα) alone or in combination. Tunicamycin-induced ER stress caused liver injury; promoted the accumulation of cholesterol and triglycerides; inhibited the expression of LXRα, ABCA1 and ABCG1 in the livers of mice, thus reducing serum high-density lipoprotein (HDL)-C, low-density lipoprotein (LDL)-C, total cholesterol and triglyceride levels; however, LXR-623 could attenuate ER stress and reverse these changes. We also obtained the same results in Huh-7 and THP-1 cells. ER stress induced by tunicamycin could clearly be reversed by activating LXRα because it promoted cholesterol efflux by enhancing the expression of ABCA1 and ABCG1 in hepatocytes and macrophages, contributing to attenuation of the development of AS.

## 1. Introduction

Atherosclerosis (AS) is the most common cardiovascular disease, and hyperlipidemia, the presence of abnormally high plasma cholesterol and triglyceride levels, is a common risk factor for AS [1]. Cholesterol is mainly transported in the form of lipoproteins in blood circulation; research has shown that low-density lipoprotein (LDL) is the main factor that causes AS [2], while high-density lipoprotein (HDL) has a preventive effect against AS by reversing the transport of cholesterol from peripheral cells back to the liver; both the liver and macrophages participate in the metabolism of HDL and LDL [3].

The liver is associated with the lipid metabolism including triglycerides, cholesterol and plasma lipoproteins [4,5]. In cholesterol metabolism in the liver, reverse cholesterol transport (RCT) plays a crucial role in cholesterol homeostasis in vivo and has been recognized as the main mechanism by which HDL prevents AS [6]. During RCT, apolipoprotein A1 (ApoA1), mainly synthesized by the liver and small intestine, accepts phospholipids and cholesterol efflux from the liver (macrophages) through ABC subfamily A member 1 (ABCA1) to form nascent HDL, which continuously accepts cholesterol transported through ATP binding cassette (ABC) subfamily G member 1 (ABCG1) from the peripheral tissue and macrophages to form mature HDL. Moreover, scavenger receptor class B type 1 (SR-BI), an HDL receptor on liver cell membranes, selectively takes up HDL cholesterol; thus, cholesterol is transported into the liver and modified into bile acids and other substances, excluding cholesterol transported out of the body [6]. Therefore, disordered cholesterol metabolism in the liver and macrophages may promote the development of AS.

The endoplasmic reticulum (ER) participates in protein and lipid metabolism [7]. ER stress, which causes the unfolded protein response (UPR), can be caused by various disturbances, including infection, calcium regulation instability, and redox regulation disturbance, and is harmful to cells [8,9]. It was largely proved that ER stress plays a vital role in maintaining cholesterol metabolism and homeostasis in macrophages [10,11]. For example, Yan et al. found that ER stress can regulate the lipid catabolism of macrophages by promoting cholesterol uptake, inhibiting cholesterol efflux, and modulating the expression of related transporters [12]. However, inhibition of ER stress by naringenin can enhance the expression of ABCA1 and reverse the effect of ER stress, contributing to the efflux of cholesterol from macrophages and attenuating the development of AS [13]. In fact, ER stress can also modulate lipid metabolism in the liver. Recent studies have uncovered the contribution of ER stress in the regulation of hepatic steatosis and the cellular response to lipotoxic stress [14], which is related to the remarkable increase in the hepatic triglyceride content due to ER stress and the reduced secretion of plasma lipoproteins in mice [15]. Furthermore, ER stress led to increased cell surface hepatic LDLR expression and reduced circulating LDL concentrations in cultured Huh-7 and HepG2 hepatocytes, helping to lower LDL cholesterol levels [16]. However, the influence of ER stress on cholesterol efflux in hepatocytes is not clear.

As mentioned above, ER stress is involved in regulating cholesterol metabolism in the liver and macrophages, both of which are directly linked to AS development. In addition, as a key modulator in the RCT process, LXRα occupies an extremely important position in the process of cholesterol metabolism in the liver and macrophages by regulating the transcription of ABCA1 and ABCG1, which belong to the nuclear hormone receptor superfamily and have two homologous subtypes (LXRα and LXRβ) [17]. However, whether ER stress modulates cholesterol efflux by LXRα in hepatocytes and macrophages needs to be revealed. Therefore, in this study, we intended to explore the connection between ER stress induced by tunicamycin and LXRα in hepatocytes and macrophages and clarify the possible mechanism and their roles in AS.

## 2. Materials and Methods

### 2.1. Animals

C57BL/6 mice were purchased from the Laboratory Animal Science Center of Nanchang University. Six-month-old male mice were randomly divided into the following 4 groups, with 6 mice in each group: (1) mice given an intraperitoneal injection of DMSO diluted in PBS at the same concentration as the model group, used as a normal control group (C); (2) mice intraperitoneally injected with 1 mg/kg tunicamycin (TM) dissolved in DMSO and diluted with PBS for 48 h; (3) mice intraperitoneally injected with 15 mg/kg LXR-623 (L, agonist of LXRα, Cat.No. HY-10629; MedChemExpress, Monmouth Junction, NJ, USA) dissolved in DMSO and diluted with PBS for 48 h; (4) combined treatment group (TM + L), the mice in which were injected intraperitoneally with 1 mg/kg TM dissolved in DMSO and diluted with PBS and 15 mg/kg LXR-623 by injection 2 h later with treatment sustained for 48 h. During treatment, the volume of DMSO injected into each group of mice was equal. Ultimately, the mice were sacrificed after 12 h of fasting. Plasma was collected for the determination of serum aspartate aminotransferase (AST), alanine aminotransferase (ALT), triglyceride, total cholesterol, high-density lipoprotein and low-density lipoprotein levels. Liver tissues were collected and fixed in 4% paraformaldehyde for H&E staining, and the remaining tissues were frozen at −80 °C for tissue triglyceride and total cholesterol determination and Western blot analysis. The experiment was approved by the Animal Protection and Use Ethics Committee of Nanchang University (NDSYDWLL-201761).

### 2.2. Cell Culture

Huh-7 and THP-1 cells were purchased from the Cell Bank of the Type Culture Collection of the Chinese Academy of Sciences (Shanghai, China). Huh-7 cells were cultured in Dulbecco’s modified Eagle’s medium (DMEM) containing penicillin (100 U/mL), streptomycin (0.1 mg/mL), and 10% FBS. THP-1 cells were cultured in RPMI 1640 medium containing the same ingredients as described above. Both cell lines were kept in a humidified chamber at 37 °C with 5% CO_2_. To differentiate THP-1 monocytes into macrophages, they were exposed to 100 nmol/L phorbol-12-myristate 13-acetate (PMA, Cat.No. HY-18739; MedChemExpress, Monmouth Junction, NJ, USA) for 48 h.

### 2.3. Serum Metabolite Profile Analysis

After the mice had been sacrificed, we centrifuged the plasma to collect serum and stored the serum at 4 °C. AST, ALT, triglyceride, total cholesterol, HDL-C and LDL-C levels were analyzed within 24 h using specific kits (Nanjing Jiancheng Bioengineering Research Institute, Nanjing, China) according to the manufacturer’s instructions. All procedures were performed on ice.

### 2.4. Liver Triglyceride and Total Cholesterol Assay

Liver triglyceride and total cholesterol levels were detected using a commercial assay kit (Nanjing Jiancheng Bioengineering Research Institute, Nanjing, China) according to the manufacturer’s instructions.

### 2.5. H&E Staining

The collected fresh liver tissues were fixed in 4% paraformaldehyde for 48 h, dehydrated, cleared and embedded in paraffin, after which they were subjected to tissue sectioning. The slices were dehydrated, stained with hematoxylin for 10 min, washed with ddH_2_O, differentiated with 10% hydrochloric acid ethanol for 2 s, washed again with ddH_2_O halfway, treated with 1% ammonia inverse blue for 2 s, washed with ddH_2_O and stained with eosin for 5 min. The sections were then dehydrated and fixed on glass slides with neutral resin. An image analysis system was used to collect images and take pictures under a microscope (Olympus IX71).

### 2.6. Oil Red O Staining

After cells cultured in 12-well plates had been treated, the culture medium was decanted, washed three times with PBS, fixed with 4% paraformaldehyde for 30 min, and rinsed with 60% isopropanol for 20 s. An oil red O dye solution (Cat. No. G1262; Solarbio Life Science, Beijing, China) was used to cover the bottom plate and stain the cells. After staining for 10 min, cells were differentiated with 60% isopropyl alcohol until the background became colorless, and excess dye was removed. After counterstaining, hematoxylin was used to stain the nuclei for 30 s, which were then washed with PBS. A microscope with a CCD camera was used to observe and capture images (Olympus IX71). The Image J 7.6 software was used to analyze these results. In brief [18], we opened the file of cell photos using Image J software, then clicked “image” and selected “adjust”; next, we selected “Color Threshold”. In this interface, we adjusted saturation and brightness of the photo to turn the area of the stained lipid droplets. After that, we clicked “original” and “Filtered” to compare the original image with the changed image and checked whether the areas of lipid droplets were the same. Finally, we clicked “Analyze” and selected “Measure” to measure the area of lipid droplets. The cell numbers in each picture were counted for the difference of cell numbers in different pictures. The average lipid accumulation in each picture was equal to area/cell numbers [18].

### 2.7. Filipin Staining

Equal amounts of cells were cultured on sterile slides in 24-well plates. When the cell density in each well reached 70%, the cells were treated with drugs. Subsequently, the medium was gently removed, and the cells were washed 3 times with PBS. Then the cells were fixed with 4% paraformaldehyde for 30 min, washed 3 times with PBS again, and incubated with 1.5 mg/mL glycine for 10 min. Finally, 500 μL of filipin (50 μg/mL, Cat. No. FF8614; Hefei Bomei Biotechnology Co., Ltd., Hefei, China) was added to each well and incubated for 2 h, after which the experimental results were observed and recorded using a fluorescence microscope (magnification, 10×; Olympus IX71).

### 2.8. Western Blot Analysis

Cells were collected into a centrifuge tube. RIPA lysate mixed with protease inhibitor and phosphatase inhibitor was added to lyse the cells to obtain the total proteins. To prepare liver protein lysate, the liver tissue with added RIPA lysate mixed with protease inhibitor and phosphatase inhibitor was homogenized with a tissue homogenizer and centrifuged at 12,000× *g* and 4 °C, after which the supernatant was collected. The protein concentration was determined using the BCA assay (CWBIO, Beijing, China). Then, an equal amount of protein from each group was electrophoresed on an 8% or 10% gel. We transferred all the proteins to a PVDF membrane that was blocked with 5% skim milk for 1 h. Subsequently, the PVDF membrane was soaked in primary antibodies to detect the expression of the key ER stress molecules CHOP (Cat. No. AP11955B; dilution, 1:2000; ABGENT, Suzhou, China) and GRP78 (Cat. No. 66574-1-lg; dilution, 1:50,000; ProteinTech Group, Inc., Wuhan, China), ABCA1 (Cat. No. D155299; dilution, 1:1000; Sangon Biotech Co., Ltd., Shanghai, China), ABCG1 (Cat. No. D160810; dilution, 1:2000; Sangon Biotech Co., Ltd., Shanghai, China), LXRα (Cat. No. D154187; dilution, 1:2000; Sangon Biotech Co., Ltd., Shanghai, China), HMGCR (Cat. No. D154106; dilution, 1:2000; Sangon Biotech Co., Ltd., Shanghai, China) and GAPDH (Cat. No. HRP-60004; dilution, 1:50,000; ProteinTech Group, Inc., Wuhan, China). After washing with TBST, the secondary antibody was added to the primary antibody and incubated with the proteins. Finally, an ECL chemiluminescence kit (Cat. No. WLA006; Wanlei Biotechnology Co., Ltd., Shengyang, China) was used to visualize the blots. Every result was collected at least three times and analyzed by Image Lab.

### 2.9. Statistical Analysis

All results were analyzed by GraphPad Prism 6.0. The differences between each group were assessed by one-way analysis of variance (ANOVA) and the Tukey post-hoc test. The results were repeated at least three times; *p* < 0.05 indicated statistical significance.

## 3. Results

### 3.1. LXR-623 Can Reverse Liver Injury Caused by ER Stress

**Figure 1:**
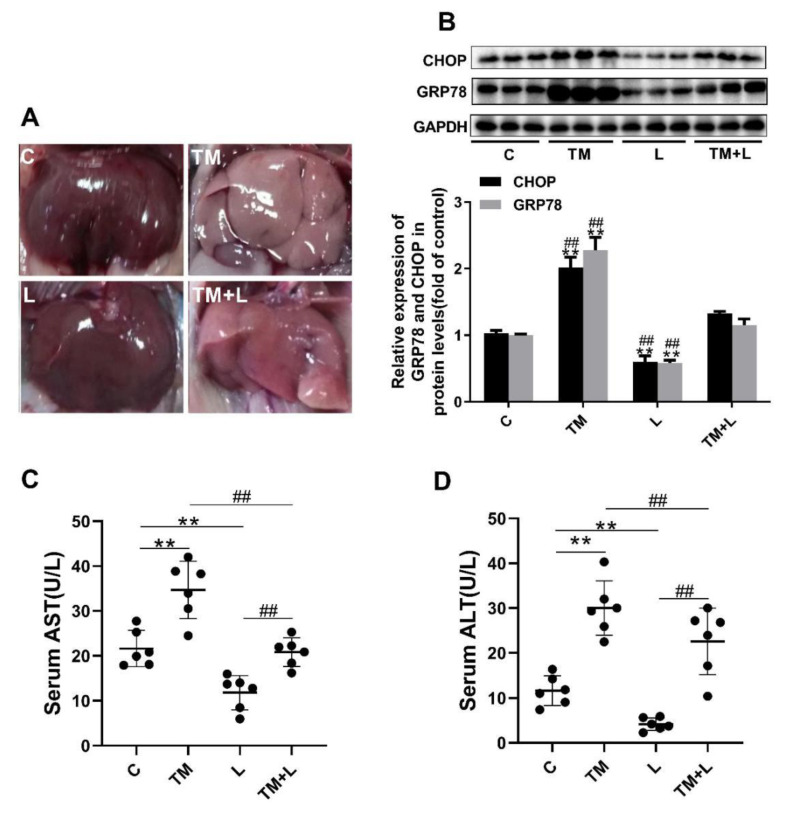
Tunicamycin-induced endoplasmic reticulum (ER) stress damaged hepatic function, and LXR-623 reversed this effect. Six-month-old mice were injected intraperitoneally with vehicle, 1 mg/kg tunicamycin and 15 mg/kg LXR-623. The livers were collected 48 h later. (**A**) Fresh liver morphology at harvest. (**B**) Expression levels of the ER stress indicator proteins CHOP and GRP78 in the liver. (**C**) Serum aspartate aminotransferase (AST) level. (**D**) Serum alanine aminotransferase (ALT) level. C, control; TM, tunicamycin; L, LXR-623; TM + L, tunicamycin + LXR-623. Data are shown as the mean ± SD (*n* = 6), ** *p* < 0.001 versus the C group. ^##^ *p* < 0.001 versus the TM + L group.

To study the effect of tunicamycin-induced ER stress and LXR-623 on hepatic function, we injected tunicamycin, LXR-623, or vehicle separately or a combination of tunicamycin and LXR-623 into the abdominal cavities of mice. The results collected after 48 h of treatment showed that the livers of the mice treated with tunicamycin (TM group) were more yellow than those of the control group (C). However, after treatment with LXR-623 (L group), the livers of the mice did not change significantly. Although the livers of mice treated with tunicamycin and LXR-623 (TM + L) were also significantly more yellow than those of the control group, the degree of discoloration was clearly improved compared with that of the tunicamycin group (Figure 1A). After that, we tested the hepatic ER stress level after treatment with tunicamycin and LXR-623. The results showed that two molecular indicators of ER stress, CHOP and 78 kDa glucose-regulated protein (GRP78), were upregulated in the group treated with tunicamycin. After LXR-623 treatment, the expression of CHOP and GRP78 in mice was downregulated. In the TM + L group, the expression levels of the two proteins were between those in the tunicamycin and LXR-623 groups (Figure 1B). These data indicated that tunicamycin effectively induced ER stress after 48 h and that LXR-623 reversed the effect of tunicamycin. In addition, we tested the levels of AST and ALT, which are two important indicators of hepatic function, in mouse serum. The final results showed that the serum AST and ALT levels were higher than those in the control group after mice were treated with tunicamycin, and the levels of both indicators were lower than those in the control group after LXR-623 treatment. In the TM + L group, the same results observed before were found, as the levels of AST and ALT were between those in the other two groups (Figure 1C,D). The above data indicated that tunicamycin induced ER stress and impaired hepatic function and that the LXR agonist LXR-623 reversed these effects.

### 3.2. LXR-623 Can Reduce Liver Lipid Accumulation Caused by ER Stress

**Figure 2:**
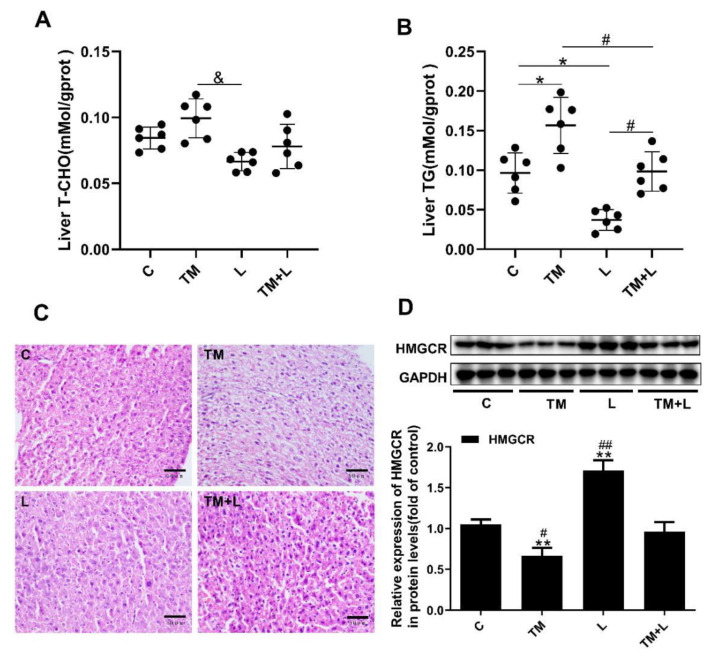
Tunicamycin-induced ER stress caused liver lipid accumulation, and LXR-623 had the opposite effect. (**A**) Liver total cholesterol level. (**B**) Liver triglyceride level. (**C**) H&E staining of the liver. (**D**) Expression levels of HMGCR in the liver. C, control; TM, tunicamycin; L, LXR-623; TM + L, tunicamycin + LXR-623. Data are shown as the mean ± SD (*n* = 6), * *p* < 0.05 or ** *p* < 0.001 versus the C group. ^#^ *p* < 0.05 or ^##^ *p* < 0.001 versus the TM + L group. ^&^ *p* < 0.05 versus L group.

Changes in liver color indicate that lipid accumulation in the liver may have occurred after tunicamycin treatment [15]. Therefore, the total cholesterol and triglyceride content in the liver was detected. The results showed that tunicamycin significantly increased the amount of total triglyceride in the liver, while the LXR-623 group showed a prominent decrease in the amount of both, and the amount of triglyceride in the TM + L group was between that in the tunicamycin and LXR-623 groups. However, the results of cholesterol content were not significantly different, which may be due to an insufficient tunicamycin treatment time (48 h) or other reasons (Figure 2A,B). The H&E staining results also showed that more lipid droplets accumulated in the livers of mice treated with tunicamycin compared to mice in the control group. In contrast, fewer fat droplets accumulated in the livers of mice in the LXR-623 group, and the hepatic morphology was not significantly different than that in the control group. The degree of hepatic lipid accumulation in the TM + L group was significantly lower than that in the tunicamycin group (Figure 2C). Moreover, we also detected the protein expression of HMGCR in the liver. The expression of HMGCR by liver cells decreased after tunicamycin treatment but was increased in the LXR-623 group. The TM + L group showed HMGCR expression level was between that in the tunicamycin group and LXR-623 group (Figure 2D). These findings showed that hepatic lipid accumulation exhibits a negative feedback mechanism, reducing cholesterol synthesis to maintain lipid metabolism balance. The above results indicated that the administration of tunicamycin for 48 h could induce hepatic lipid accumulation and that LXR-623 could inhibit this effect.

### 3.3. LXR-623 Reduced the Effect of ER Stress on the Inhibition of Hepatic Cholesterol Efflux

**Figure 3:**
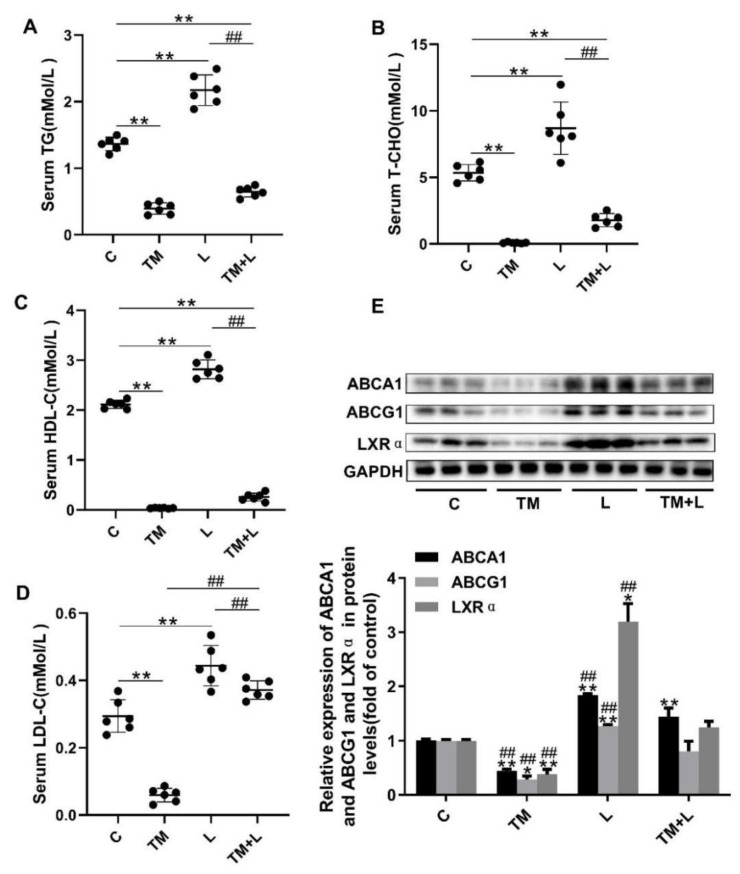
LXR-623 inhibited the effect of ER stress on hepatic lipid efflux. (**A**) Serum triglyceride levels. (**B**) Serum total cholesterol level. (**C**) Serum high-density lipoprotein (HDL) cholesterol level. (**D**) Serum low-density lipoprotein (LDL) cholesterol level. (**E**) Expression levels of ABCA1, ABCG1 and LXRα in the liver. C, control; TM, tunicamycin; L, LXR-623; TM + L, tunicamycin + LXR-623. Data are shown as the mean ± SD (*n* = 6), * *p* < 0.05 or ** *p* < 0.001 versus the C group. ^##^ *p* < 0.001 versus the TM + L group.

To further investigate the cause of liver lipid accumulation by tunicamycin, serum HDL-C, LDL-C, total cholesterol and triglyceride levels were measured. The results showed that tunicamycin significantly downregulated the serum levels of these four indicators compared with their levels in the control group, while they were increased in the LXR-623 group, and the TM + L group showed levels that were between those of the tunicamycin and LXR-623 groups (Figure 3A–D). Next, the expression levels of ABCA1, ABCG1 and LXRα were measured by Western blotting. Then we found that tunicamycin remarkably decreased their expression levels compared with those in the control group, while their expression levels increased after treatment with LXR-623. The expression levels of these proteins in the TM + L group were between those in the tunicamycin and LXR-623 groups (Figure 3E). The above results indicated that tunicamycin inhibited hepatic cholesterol efflux and that LXR-623 had the opposite effect.

### 3.4. LXR-623 Inhibited ER Stress-Induced Cholesterol Accumulation in Huh-7 Cells

**Figure 4:**
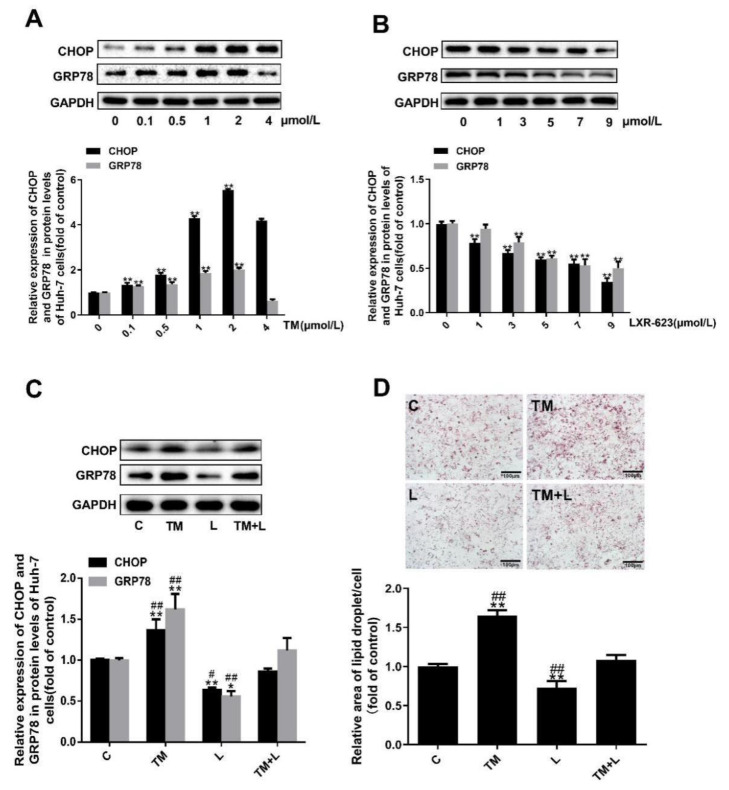
Tunicamycin-induced ER stress caused lipid accumulation in Huh-7 cells, and LXR-623 mitigated this effect. (**A**,**B**) Huh-7 cells were treated with various doses of tunicamycin (0, 0.1, 0.5, 1, 2, 4 μmol/L) and LXR-623 (0, 1, 3, 5, 7, 9 μmol/L). Expression levels of CHOP and GRP78 in Huh-7 cells. (**C**) Expression levels of CHOP and GRP78 in Huh-7 cells in 4 treatment groups. (**D**) Oil red O staining of Huh-7 cells. C, control; TM, tunicamycin; L, LXR-623; TM + L, tunicamycin + LXR-623. Data are shown as the mean ± SD (*n* = 3), * *p* < 0.05 or ** *p* < 0.001 versus the 0 or C group. ^##^ *p* < 0.001 versus the TM + L group.

To examine the above results in greater depth, we treated Huh-7 human liver cancer cells with tunicamycin and LXR-623 and determined the lipid content of the cells with oil red O and filipin staining. To find the optimal concentration for drug treatment, the concentration of tunicamycin was increased from 0 to 4 μmol/L, and that of LXR-623 was increased from 0 to 9 μmol/L. It was found that tunicamycin could upregulate the expression levels of GRP78 and CHOP in a dose-dependent manner from 0 to 2 μmol/L (Figure 4A). However, from the results of Figure 4B, LXR-623 downregulated the expression of both proteins in a dose-dependent manner. Based on a combination of the final results with the drug concentrations used in other studies, the final tunicamycin and LXR-623 treatment concentrations were 2 μmol/L and 9 μmol/L, respectively.

Subsequently, as in the treatment of mouse livers, we also divided the cells into four groups, and the expected results were observed. Tunicamycin upregulated expression of the chaperone proteins GRP78 and CHOP and increased the total lipid and cholesterol levels. LXR-623 downregulated the expression of GRP78 and CHOP and reduced the lipid and cholesterol content of the cells. Compared with the tunicamycin group, in cells treated with the two drugs together, the expression of GRP78 and CHOP was downregulated, and the lipid levels in the cells were obviously reduced. In contrast, compared with the LXR-623 group, these values were increased (Figure 4C,D). Obviously, these data indicated that ER stress caused intracellular lipid accumulation and that LXR-623 inhibited this effect.

### 3.5. LXR-623 Inhibited the Decrease in Cholesterol Efflux Caused by ER Stress in Huh-7 Cells

**Figure 5:**
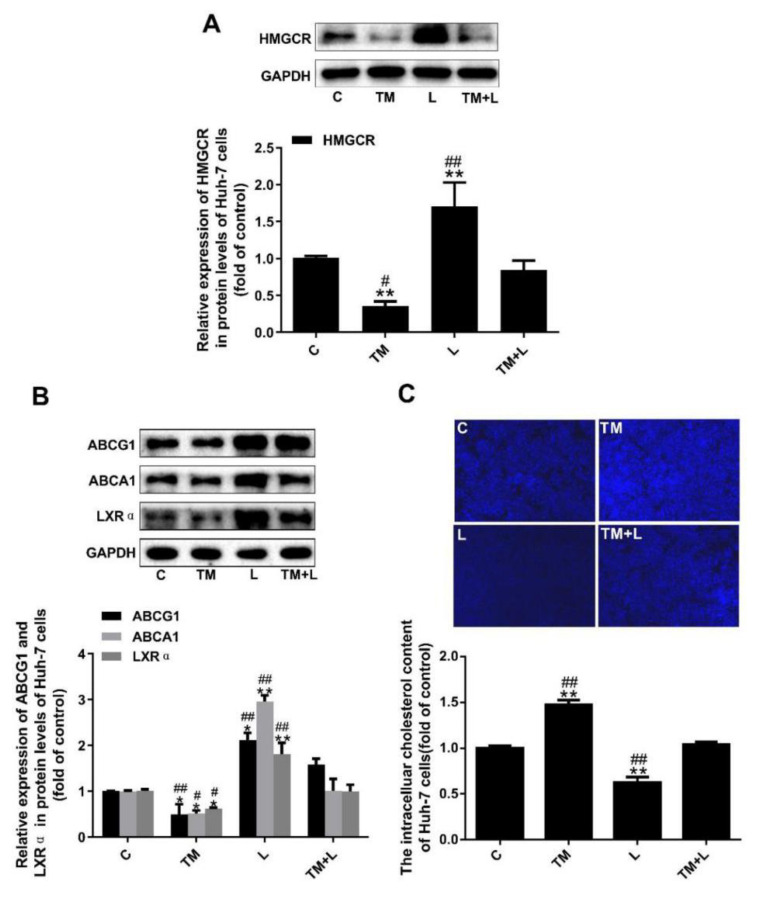
Tunicamycin-induced ER stress reduced cholesterol efflux in Huh-7 cells, and LXR-623 inhibited this effect. (**A**) Expression levels of HMGCR in Huh-7 cells. (**B**) Expression levels of ABCA1, ABCG1 and LXRα in Huh-7 cells. (**C**) Intracellular cholesterol measured by filipin staining. C, control; TM, tunicamycin; L, LXR-623; TM + L, tunicamycin + LXR-623. Data are shown as the mean ± SD (*n* = 3), * *p* < 0.05 or ** *p* < 0.001 versus the C group. ^#^ *p* < 0.05 or ^##^ *p* < 0.001 versus the TM + L group.

In this study, we measured HMGCR expression and found that tunicamycin reduced HMGCR expression, but LXR-623 upregulated HMGCR expression. Moreover, examination of the TM + L group showed that LXR-623 reduced the effect of tunicamycin on cholesterol efflux in Huh-7 cells (Figure 5A). As before, to investigate the effects of tunicamycin and LXR-623 on cholesterol efflux in Huh-7 cells, we detected the expression of ABCA1, ABCG1 and LXRα and the cellular cholesterol level and found that tunicamycin downregulated ABCA1, ABCG1 and LXRα expression and intracellular cholesterol accumulation (Figure 5B). However, the LXR-623 group showed the opposite results, and those in the TM + L group were between those in the tunicamycin and LXR-623 groups (Figure 5C).

### 3.6. LXR-623 Inhibited ER Stress-Induced Cholesterol Accumulation in THP-1 Macrophages

**Figure 6:**
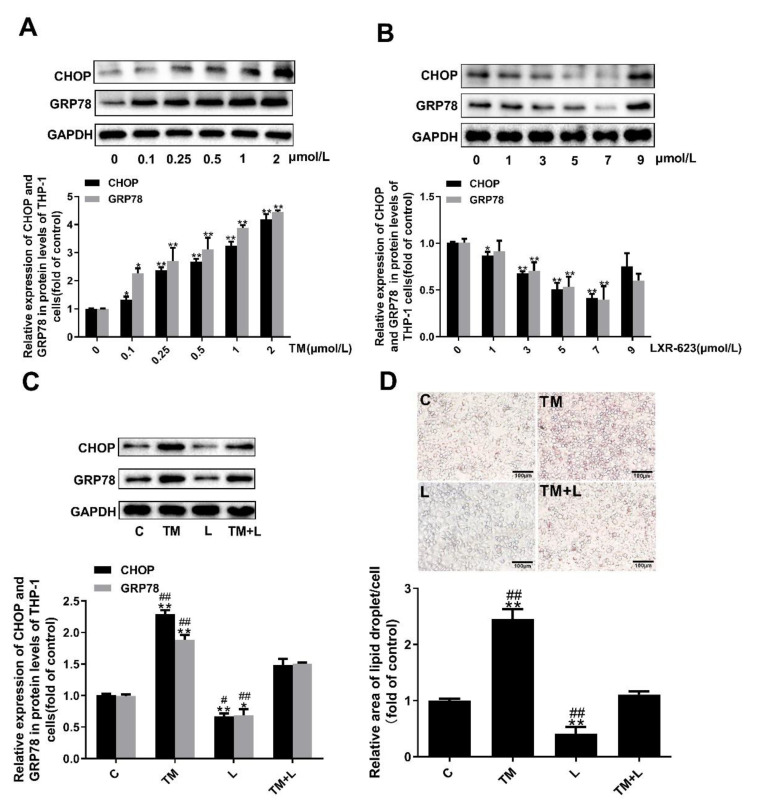
Tunicamycin-induced ER stress caused lipid accumulation in THP-1 macrophages, and LXR-623 inhibited this effect. (**A**,**B**) THP-1 macrophages were treated with various doses of tunicamycin (0, 0.1, 0.25, 0.5, 1, 2 μmol/L) and LXR-623 (0, 1, 3, 5, 7, 9 μmol/L). Expression levels of CHOP and GRP78 in THP-1 macrophages. (**C**) Expression levels of CHOP and GRP78 in THP-1 macrophages in 4 different treatment groups. (**D**) Oil red O staining of THP-1 macrophages. C, control; TM, tunicamycin; L, LXR-623; TM + L, tunicamycin + LXR-623. Data are shown as the mean ± SD (*n* = 3), * *p* < 0.05 or ** *p* < 0.001 versus the 0 or C group. ^#^ *p* < 0.05 or ^##^ *p* < 0.001 versus the TM + L group.

As with Huh-7 cells, we treated THP-1 macrophages with tunicamycin and LXR-623 and determined the lipid content of the cells with oil red O and filipin staining. Similarly, we increased the concentration of tunicamycin from 0 to 2 μmol/L and increased LXR-623 from 0 to 9 μmol/L (Figure 6A,B). Finally, 2 μmol/L and 7 μmol/L were selected as the tunicamycin and LXR-623 treatment concentrations, respectively. Then, we also divided THP-1 macrophages into four groups and found the same results shown by the liver and Huh-7 cells. Tunicamycin increased the expression of GRP78 and CHOP and increased the lipid levels. LXR-623 reduced the expression of GRP78 and CHOP and reduced the lipid content of the cells. The TM + L group showed levels between those of the TM and LXR-623 groups (Figure 6C,D). These data indicated that lipid accumulation in THP-1 cells caused by ER stress was suppressed by LXR-623.

### 3.7. LXR-623 Inhibited the Decrease in Cholesterol Efflux Caused by ER Stress in THP-1 Macrophages

**Figure 7:**
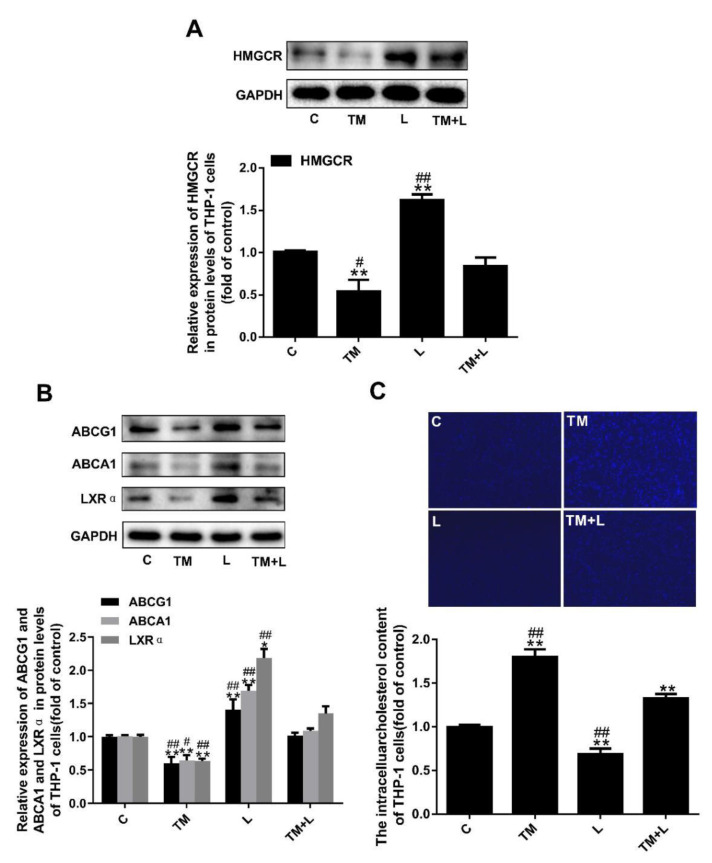
Tunicamycin-induced ER stress reduced cholesterol efflux in THP-1 macrophages, and LXR-623 inhibited this effect. (**A**) Expression levels of HMGCR in THP-1 macrophages. (**B**) Expression levels of ABCA1, ABCG1 and LXRα in THP-1 macrophages. (**C**) Filipin staining of THP-1 macrophages. C, control; TM, tunicamycin; L, LXR-623; TM + L, tunicamycin + LXR-623. Data are shown as the mean ± SD (*n* = 3), * *p* < 0.05 or ** *p* < 0.001 versus the C group. ^#^ *p* < 0.05 or ^##^ *p* < 0.001 versus the TM + L group.

Similar to the effects of tunicamycin treatment of Huh-7 cells, tunicamycin reduced HMGCR expression, which was increased in the LXR-623 group. The effects on the TM + L group were between those on the tunicamycin group and LXR-623 group (Figure 7A). Finally, to study the effect of the two drugs on cholesterol outflow from THP-1 macrophages, we examined the expression levels of ABCA1, ABCG1 and LXRα and the cellular cholesterol level, and the results demonstrated that tunicamycin decreased the expression of ABCA1, ABCG1 and LXRα, while LXR-623 upregulated their expression. The levels in the TM + L group were between those in the tunicamycin group and LXR-623 group (Figure 7B). In addition, after LXR-623 treatment, intracellular cholesterol accumulation was significantly reduced (Figure 7C).

## 4. Discussion

ER stress is an important factor that affects lipid metabolism, mainly the metabolism of cholesterol and triglycerides, and the source of many metabolic and cardiovascular diseases [19,20,21]. The liver is the main organ responsible for lipid metabolism in the body. Much evidence suggests that ER stress can cause liver lipid accumulation and even directly lead to liver injury [15,20,22]. Lee et al. demonstrated the role of ER stress in upregulating the formation of new lipid droplets in Huh-7 cells [19]. Furthermore, our results indicated that the activation of ER stress led to liver injury and obvious adipose tissue morphology degeneration. Meanwhile, in vitro, both the number of lipid droplets and cholesterol content in Huh-7 and THP-1 cells were increased after tunicamycin treatment. Clearly, ER stress could obviously lead to the accumulation of cholesterol and triglycerides in hepatocytes and macrophages; however, the detailed mechanism needs to be further explored.

LXRα plays a crucial role in the process of cholesterol metabolism process in the liver and macrophages and has also been shown to play a vital role in ER stress [23]. For example, Ge et al. demonstrated that betaine ameliorated hepatic lipid accumulation and inflammation through restoring LXRα and PPARα expression and alleviating ER stress in fructose-fed rats—an experiment which indicated that only LXRα participated in ER stress [24]. Furthermore, Zhao et al. confirmed that LXRα degradation is a key factor that induces ER stress stimulated by IFN-γ depending on the PIAS1/STAT1 pathway in macrophages [25]. However, the detailed mechanism is not fully clear. LXRα plays a pivotal role in the transcriptional control of lipid metabolism including the cholesterol, fatty acid and phospholipids metabolism [26]. In this study, we firstly discovered that tunicamycin-induced ER stress could regulate the cholesterol efflux by blocking the expression of LXRα, ABCA1 and ABCG1 in hepatocytes and macrophages; LXR-623, an agonist of LXRα, could attenuate tunicamycin-induced ER stress. Obviously, our findings again clearly confirmed that LXRα plays a key role in ER stress and further revealed that this role may be related to ABCA1 and ABCG1, and cholesterol efflux. LXRα is a transcription factor that can regulate the expression of ABCA1 and ABCG1 [27,28]. Many studies have shown that the activation of LXRα promotes the expression of ABCA1 and ABCG1 [29,30], and we also verified that LXRα could upregulate ABCA1 and ABCG1 in hepatocytes and macrophages treated with LXR-623. RCT is essential for cholesterol metabolism homeostasis in the body [31]. During RCT, both ABCA1 and ABCG1 can mediate cholesterol efflux to the apoA1 receptor and nascent HDLs in hepatocytes [32]. Once the expression level of LXRα was decreased by tunicamycin, the expression levels of ABCA1 and ABCG1 would also be downregulated, attenuating the RCT process from macrophages or extra-hepatic tissues, altering the homeostasis of cholesterol in hepatocytes and macrophages and causing cholesterol accumulation; in contrast, the activation of LXRα induced by LXR-623 reversed the accumulation of cholesterol and triglycerides in hepatocytes and macrophages. The ER is involved in lipid metabolism; the destruction of lipid homeostasis can trigger ER stress [33,34]. For example, in vivo and in vitro studies demonstrate that saturated fatty acids promote ER stress [35]. Moreover, our previous results verified that cholesterol deposition could cause endothelial dysfunction by activating ER stress [36,37]. In this study, we proved that tunicamycin-induced ER stress elevated both the cholesterol and triglyceride content in Huh7 and THP-1 cells, and LXR-623 reversed these changes. However, in the livers of mice, we also observed similar results regarding cholesterol content, but these results were not significantly different, which may be due to an insufficient tunicamycin treatment time (48 h) or other reasons. Although both fat and cholesterol induce ER stress, the regulation of ER stress is mainly caused by cholesterol because LXR-623 mainly modulates cholesterol efflux by increasing the expression of ABCA1 and ABCG1. Some studies have demonstrated that LXRα can promote the biosynthesis of fatty acids and triglycerides; therefore, agonists of LXRα can increase the biosynthesis of fatty acids and triglycerides, leading to hepatosteatosis [38,39]. However, in this study, LXR-623 treatment did not lead to lipid accumulation but instead lowered lipid accumulation in hepatocytes and macrophages. Hence, the activation of ER stress is not associated with LXR-623 by the accumulation of fatty acids and triglycerides; in contrast, the decrease in lipids in hepatocytes and macrophages was mainly related to ER stress because the inhibition of ER stress could attenuate lipid metabolism [19,20,21]. Moreover, the expression of HMGCR was effectively reduced in tunicamycin-induced ER stress, but LXR-623 could promote its expression, constituting a negative feedback mechanism that decreases the biosynthesis of cholesterol.

HDL, which is formed in the RCT process, is a good predictor of AS. In contrast, LDL is a poor predictor of AS. In this study, we demonstrated that tunicamycin-induced ER stress could lower serum HDL levels; however, LXR-623 reversed these alterations. Interestingly, tunicamycin-induced ER stress could also lower serum LDL levels; however, LXR-623 reversed these alterations. ER stress is a factor that promotes AS [40,41]. Therefore, HDL may be decreased in the process of ER stress. However, LXR-623 could block ER stress, leading to an increase in HDL. In addition, serum LDL was decreased because ER stress leads to increased cell surface hepatic LDLR expression, which may promote LDL intake, and then reduces serum LDL concentrations in mice [16]. Although both serum HDL and LDL were decreased, the decrease in LDL promoted ER stress in the liver, causing the accumulation of cholesterol, which continuously blocked RCT and decreased the HDL level, finally leading to the development of AS.

## 5. Conclusions

Taken together, these findings show that ER stress induced by tunicamycin can be reversed by activating LXRα because it can promote cholesterol efflux by enhancing the expression of ABCA1 and ABCG1 in hepatocytes and macrophages, contributing to attenuating the development of AS. However, in this study, since we aimed to investigate cholesterol metabolism of hepatocytes and macrophages, the mice were only treated for 2 days, and it needed more time to study the development of the AS plaque.
